# miR-152 Attenuates the Severity of Lupus Nephritis Through the Downregulation of Macrophage Migration Inhibitory Factor (MIF)-Induced Expression of COL1A1

**DOI:** 10.3389/fimmu.2019.00158

**Published:** 2019-02-06

**Authors:** Jiayi Zheng, Ruru Guo, Yuanjia Tang, Qiong Fu, Jie Chen, Lingling Wu, Lin Leng, Richard Bucala, Yang Song, Liangjing Lu

**Affiliations:** ^1^Department of Rheumatology, Renji Hospital, School of Medicine, Shanghai Jiao Tong University, Shanghai, China; ^2^Department of Internal Medicine, Yale University School of Medicine, New Haven, CT, United States

**Keywords:** miR-152, macrophage migration inhibitory Factor (MIF), COL1A1, lupus nephritis, chronicity index

## Abstract

**Background:** The role of miR-152 in lupus nephritis has not been elucidated. The aim of this study was to investigate the role of miR-152 in the pathogenesis of lupus nephritis (LN).

**Methods:** miR-152 expression was detected using RT-PCR in LN tissue and normal controls. The miR-152 expression was compared with clinical parameters such as 24 h urine protein excretion level, serum creatinine, and serum complement level and SLEDAI score. The function of miR-152 was examined using human renal proximal tubular epithelial cells (HRPTE). miR-152 mimics and inhibitors were transfected to HRPTEs to ascertain the effects of miR-152.

**Results:** miR-152 expression was downregulated in LN tissue. There was an inverse correlation between miR-152 expression in LN tissue and clinical parameters like 24 h urine protein excretion levels and serum creatinine, but not serum complement levels or SLEDAI. Further analysis showed that macrophage migration inhibitory factor (MIF) was a direct target of miR-152. Downregulation of MIF through complementary binding of miR-152 inhibited the renal expression of COL1A1.

**Conclusion:** miR-152 expression was tapered in LN tissue and miR-152 expression was inversely correlated with chronicity index (CI), serum creatinine and severity of proteinuria. miR-152 may attenuate the severity of LN through the downregulation of MIF-induced expression of COL1A1. These findings suggest that miR-152 may be a potential target for the treatment of LN.

## Introduction

Systemic lupus erythematosus (SLE) is a multi-organ autoimmune disorder that typically affects women of childbearing age. It is characterized by a loss of immune tolerance and production of autoantibodies. Clinical findings of SLE vary from malar rash and mild arthralgia to life-threatening lupus nephritis (LN) and CNS lupus. Of note, renal involvement occurs in over 60% of patients with SLE and is a strong predictor of morbidity and mortality (1). LN may present with hematuria, proteinuria or an elevation in serum creatinine. Up to 10 percent of LN patients develop end-stage renal disease (2). Current treatment of LN includes immunosuppressive agents such as cyclophosphamide and mycophenolate mofetil. However, non-specific immunosuppression often leads to a variety of adverse effects, and we feel the need to explore effective targeted medication.

MicroRNAs (miRNAs) are small non-coding RNAs that downregulate the translation of protein through complementary binding to 3′-untranslated regions (3′-UTR) of the corresponding mRNA. miRNAs have been identified to participate in a variety of cellular processes including cell proliferation, differentiation, and apoptosis (3, 4) and accumulating evidence has shown that miRNAs play an important role in the pathogenesis of SLE (5). miR-152, a member of miR-148/152 family, has been reported to function as a tumor suppressor by inducing G2/M phase arrest (6), but it was only until recently that the miR-152 expression was found to be specifically elevated in the peripheral blood mononuclear cells (PBMCs) of SLE patients (7).

Macrophage migration inhibitory factor (MIF) is an innate pro-inflammatory cytokine of 12.5kDa that has been identified to be involved in multiple activities, including innate and acquired immunity (8). Polymorphisms of *MIF* gene have been linked to diseases such as systemic-onset juvenile idiopathic arthritis (9), systemic sclerosis (10), SLE (11), idiopathic pulmonary fibrosis (12), and rheumatoid arthritis (13). MIF is also confirmed to antagonize the immunosuppressive effects of glucocorticoids by counteracting the steroid induction of cytosolic IkBa, an inhibitor of NF-kB (14). Studies have shown that MIF levels are significantly elevated in patients with SLE (15), and the high serum MIF levels have been linked to SLE disease damage (SLICC/ACR index) (16). High-expression *MIF* alleles have been associated with the development of LN (11). However, the relationship between miRNAs and MIF has not been elucidated.

Though miR-152 expression has been found altered in PBMCs of SLE patients (7), no studies to date have discussed the relationship between renal miR-152 expression and the disease activity of LN. In this study, we found that miR-152 expression was significantly reduced in LN renal tissue. Further analysis showed that miR-152 downregulated COL1A1 expression in renal epithelial cells through the inhibition of MIF in renal tubular cells. We also found that reduced miR-152 expression in LN tissue was associated with higher chronicity indices (CI) on histopathological examination, higher serum creatinine levels, and higher 24 h urine protein excretion levels in LN patients. These findings indicated that miR-152 might be involved in the pathogenesis of LN and may serve as a novel biomarker for disease progression and a therapeutic target for treatment of LN.

## Materials and Methods

### Subjects

Renal tissue samples were obtained from 22 patients diagnosed with SLE at Renji Hospital who underwent percutaneous renal biopsy and were confirmed LN by a histopathological examination. All patients with LN fulfilled the American College of Rheumatology 1982 revised criteria for SLE (17).

The study was reviewed and approved by the ethics committee of Renji Hospital, Shanghai Jiao Tong University School of Medicine and the study was performed according to the terms of the Helsinki Declaration. All patients participating in this study provided signed and written informed consent.

After inclusion, patients' medical history and laboratory test results were collected. The laboratory parameters included complete blood count, serum creatinine levels, serum complement levels and 24 h urine protein excretion levels. Disease activity of the patients was measured by Systemic Lupus Erythematosus Disease Activity Index (SLEDAI). Renal biopsy were performed within 1 week of the collection of medical history and laboratory tests.

Human renal tissue controls were obtained from the non-tumorous adjacent tissues of 20 patients who underwent nephrectomy because of renal cell carcinoma. The non-tumorous adjacent tissues were dissected at least 2 cm away from the tumor border and were confirmed to be absent of tumor cells under microscopic examination. The clinical information of the subjects enrolled in this study is listed in Supplementary Tables 1, 2.

### Prediction of Possible miRNA Targeting MIF mRNA

**Figure 1:**
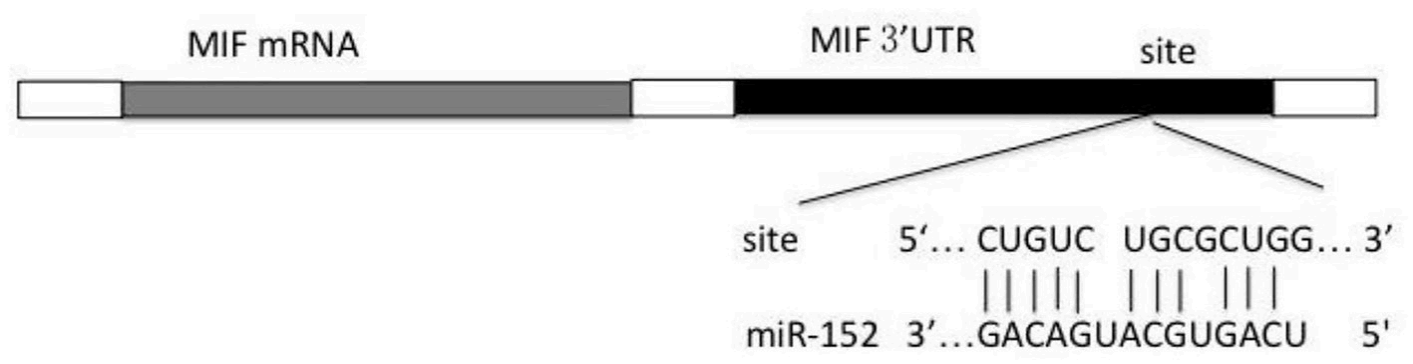
Predicted miRNA and the binding site with 3′-UTR region of MIF.

Prediction of potential binding sites of miRNA within the 3′-UTR of MIF mRNA was performed using TargetScan, PicTar, miRDB and RNA hybrid. miR-152 was found to be the possible miRNA binding 3′-UTR of MIF mRNA (Figure 1).

### Luciferase-Based Reporter Assay

The miR-152 mimics and the control vector were from GenePharma, Co. Ltd (Shanghai, China). The 3′-UTR of MIF mRNA was amplified from complementary strand of DNA using the following primers: forward 5′-AGGGTCTACATCAACTATTACGAC-3′, and reverse 5′- ACCAATTCCTCCCGCAAG-3′. The newly synthesized DNA was then cloned into psiCHECK-2 control vector (Clontech Laboratories, Inc., USA) within XhoI and NotI restriction sites. The mutations within the MIF-3′UTR were then introduced. Wild-type and mutated MIF 3′-UTR were co-transfected with miR-152 or NC to HeLa cells using Lipofectamine 2,000 (Life Technologies, USA). Cells were harvested 24 and 48 h after transfection. Firefly and Renilla Luciferase activities were measured using the Dual Luciferase Reporter 1,000 Assay System (Promega, USA) following the protocol. Firefly luciferase activity was then normalized to renilla luciferase activity.

### Extraction of Protein and RNA From Renal Tissue

The renal tissue was divided into two equal parts after being washed in PBS. One part was stored in liquid nitrogen and the other was fixed in 4% paraformaldehyde.

Samples stored in liquid nitrogen were lysed and homogenized in Buffer RLT (Qiagen Inc., USA), in case of the degradation of protein and RNA by enzymes and RNAses. The lysate was then used for protein and RNA extraction using Allprep RNA/protein mini Kit (Qiagen Inc., USA) according to its instructions.

### Real-Time PCR

mRNA sequences of RPL13A, COL1A1, TGF-β, IL-6, and CXCL10 were found in NCBI database. Primers needed to amplify the genes were listed: RPL13A forward, 5′-CCTGGAGGAGAAGAGGAAAGAGA-3′, reverse, 5′-TTGAGGACCTCTGTGTATTTGTCAA-3′; COL1A1 forward, 5′-GTGCGATGACGTGATCTGTGA-3′, reverse, 5′-CGGTGGTTTCTTGGTCGGT-3′; TGF-β forward, 5′-CAATTCCTGGCGATACCTCAG-3′, reverse, 5′-GCACAACTCCGGTGACATCAA-3′; IL-6 forward, 5′-AGCCACTCACCTCTTCAGAAC-3′, reverse, 5′-GCCTCTTTGCTGCTTTCACAC-3′; CXCL10 forward, 5′-TTCTGATTTGCTGCCTTATC-3′, reverse, 5′-CTTGGATTAACAGGTTGATTACT-3′. SYBR RT-PCR kit (Takara Bio, Dalian, China) were then used for real-time PCR. PCRs were carried out as the instructions. Each sample was analyzed in duplicate, and SYBR fluorescence was detected using real-time PCR system (Applied Biosystems Viia7, Thermo Fisher Scientific, USA). The thermal cycling conditions included 10 min at 95°C and then 40 cycles of amplification for 15 s at 95°C and 1 min at 60°C. All data were normalized to GADPH. The relative expression level of miRNAs was normalized to that of internal control by using 2^−ΔΔ*Ct*^ cycle threshold method.

Strand specific RT-PCR was used to detect the expression of MIF mRNA because of the presence of MIF-AS-1 on the complementary strand of MIF gene. First, we used Oligo 6 to design the primers needed for real-time PCR reaction: Rp13a Tag+lower 5′-ACTTGCCTCAGTTCGCTACTTTGAGGACCTCTGTGTATTTGTCAA-3′; MIF Tag +lower 5′-ACTTGCCTCAGTTCGCTACTGGAGTTGTTCCAGCCCACATTG-3′. Then primers were processed through BLAST analysis (www.ncbi.nlm.nih.gov/BLAST) to ensure the specificity of PCR. Reverse transcription were carried out using PrimeScript Reverse Transcriptase. Thermal conditions for reverse transcription were 50°C for 15 min (for reverse transcription), 85°C for 10 s (for the deactivation of reverse transcriptase) and stored at 4°C. Exonuclease-I (Thermo Fisher Scientific, USA) was then added to the system and the system was kept at 37°C for 30 min to excise the primers of cDNA. The system was then kept at 70°C for 15 min for deactivation of exonuclease I. PCRs were carried out following the procedures mentioned above.

### Cell Transfection

Human renal proximal tubular cell lines (HRPTE) were from ScienCell Research Laboratories (USA). siRNA nc, and three siRNAs targeting MIF were designed and then synthesized by GenePharma, Co. Ltd. The sequences of siRNAs are listed below: si-MIF-1 5′-CTACATCAACTATTACGACAT-3′; si-MIF-2 5′-CCTGCACAGCATCGGCAAGAT-3′; si-MIF3 5′-GCGCGCAGAACCGCTCCTACA-3′. HRPTEs were transfected with these RNAs using Lipofectamine RNAiMAX (Life Technologies, USA), according to the manufacturer's instructions.

### Western Blot Analysis

Protein from renal tissue was quantified using BCA Protein Assay Kit (Beyotime, China). Each equal amount of protein was denatured and incubated in 10% sodium dodecyl sulfate-polyacrylamide gel electrophoresis (SDS-PAGE) and transferred to polyvinylidene difluoride membranes (PVDF membranes). The blots were then incubated in TBST for an hour and probed with primary antibodies against MIF (R&D systems, USA) (1:1000) overnight at 4°C, with β-actin acting as control. Following washing with TBST (10min^*^3), the membranes were incubated in anti-rabbit HRP-IgG (Cell Signaling Technology, Inc, USA) (1:5000) for an hour. Then the membrane was washed with TBST (10min^*^3). The bands were visualized using Pierce Fast Western Blot Kits (Thermo Fisher Scientific, USA).

### Histopathological Studies of Renal Lesions

Renal tissue fixed in 4% paraformaldehyde were used for histopathological studies of LN. The frozen tissue sections were stained with hematoxylin and eosin (H&E). The renal biopsies were analyzed by a qualified pathologist who was blinded to the clinical information. Renal lesions were evaluated and graded based on the activity indices (AI) and chronicity indices (CI) proposed by Austin (18, 19).

### Immunohistochemistry and Quantitation of Tissue Staining

Immunohistochemical analysis was performed with 4 μm thick sections that had been dewaxed with xylene twice (20 min each time), and then hydrated using ethanol (100, 100, 95, and 95%, 5 min each time) and distilled water for 5 min. Endogenous peroxidase was inactivated by 3% hydrogen peroxide. Antigen retrieval was performed by heating sections in sodium citrate buffer (pH 6.0). Immunohistochemical staining was performed using the primary antibodies against MIF (R&D systems, USA, 1:1000). DAB substrate kit (BD Biosciences, USA) was then used for immunohistochemical staining, according to its protocol. Renal sections were then imaged with a microscope (Leica, Germany). Integrated optical density (IOD) value was then measured by image analysis. In addition, the number of cells labeled by immunostaining was counted under high power (X400) and was expressed as percentage of positive-staining cells. The samples were analyzed by a qualified pathologist who was blinded to the clinical information.

### Statistical Analysis

The continuous variables were presented as mean ± standard deviation and were analyzed by Student's *t*-test. Pearson's correlation and Spearman correlation analyses were performed when indicated. *P* < 0.05 was considered as statistically significant. Analyses were performed using SPSS software version 20.0 (SPSS Inc., USA) and GraphPad Prism 5.0 (GraphPad Software Inc., USA).

## Results

### The Relationship Between miR-152 Expression and Disease Activity

#### The Relationship Between miR-152 Levels and Histopathological Findings on Renal Biopsies

**Figure 2:**
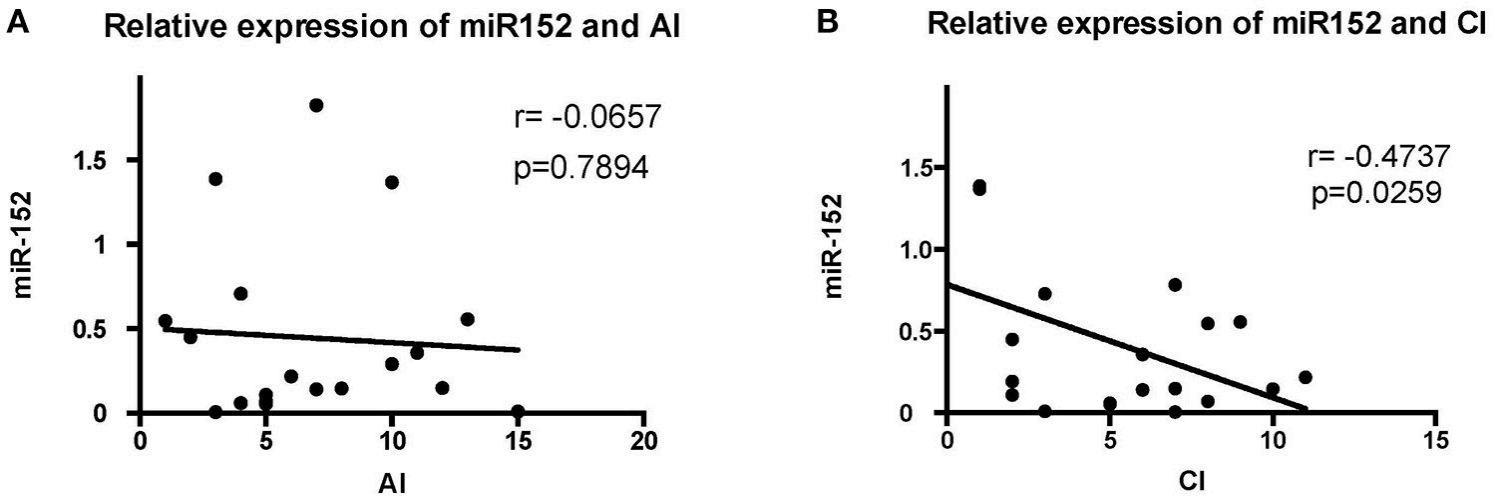
Correlation analyses of the expression of miR-152 with activity index (AI) **(A)** and chronicity index (CI) **(B)** in LN tissue. No significant correlation was found between AI and miR-152 levels; a negative correlation was found between miR-152 expression and CI.

The levels of miR-152 expression were compared with the histopathological findings on renal biopsy. Using Austin activity indices and chronicity indices, we found that levels of miR-152 expression correlated with the chronicity indices on renal biopsy (*r* = −0.4737, *p* = 0.0259). However, no correlation was found between miR-152 levels and activity indices (*r* = −0.0657, *p* = 0.7894; Figure 2).

#### The Relationship Between miR-152 Expression and Clinical Parameters and Laboratory Findings

**Figure 3:**
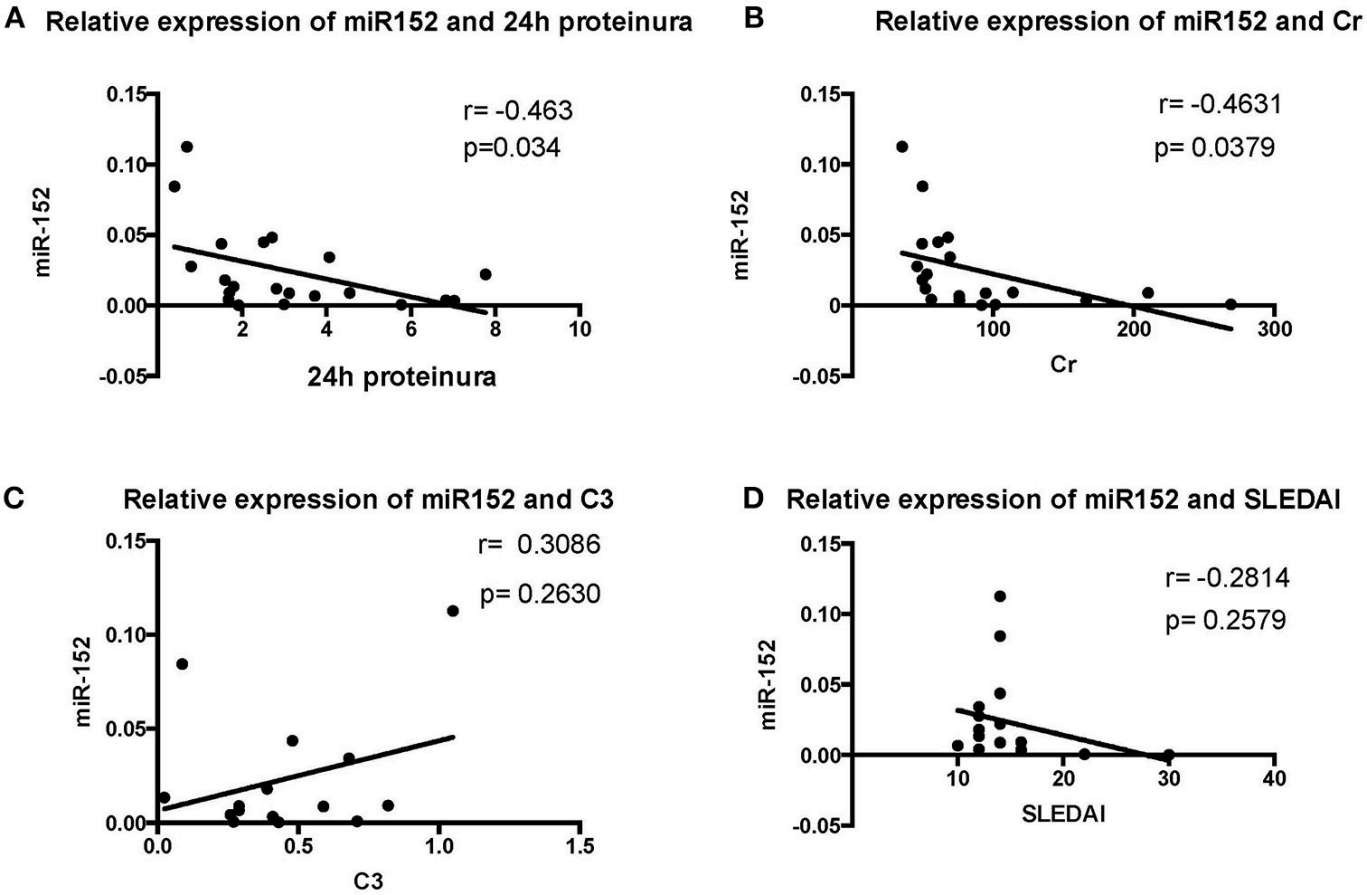
Correlation analyses between renal miR-152 expression and 24-h urine protein excretion levels **(A)**, serum creatinine levels **(B)**, serum complement C3 levels **(C)**, and the SLEDAI score **(D)** of the patients with LN.

We then analyzed the relationship between miR-152 expression in renal tissue and clinical parameters and laboratory findings of patients with LN. The 24 h urine protein excretion levels, serum creatinine levels, SLEDAI scores, and C3 levels were used for correlation analysis at the time of blood collection. The intracellular miR-152 expression was negatively correlated with serum creatinine levels (*r* = −0.4631, *p* = 0.0379) and 24 h urine protein excretion levels (*r* = −0.463, *p* = 0.034; Figure 3). Yet, no significant correlation was found between miR-152 levels and SLEDAI scores; nor was correlation observed between miR-152 levels and serum C3 levels.

### MIF Is Specifically Downregulated by the Expression of miR-152 in LN Tissue.

#### MIF Is a Direct Target of miR-152.

We employed a three-step approach to address the molecular mechanisms by which miR-152 might downregulate the expression of MIF. First, we used computational prediction programs to identify genes containing sequences that can bind to MIF 3′-UTR. Second, a luciferase-based reporter assay was performed to demonstrate that MIF was indeed a target of miR-152. Third, we compared the levels of MIF expression in primary HRPTEs treated with miR-152, miR-152 mimics, and miR-152 inhibitors to test the hypothesis that miR-152 downregulates MIF expression *in vitro*.

**Figure 4:**
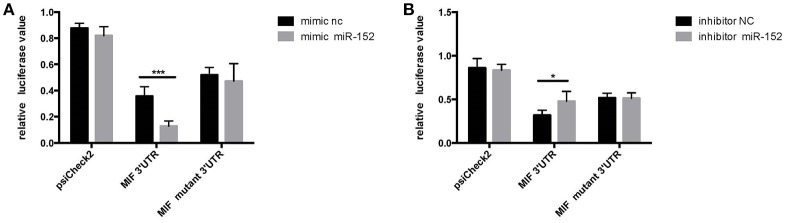
Relative luciferase activity 24 h following the co-transfection of the above reporter plasmids and a mock reporter plasmid into HeLa cells. Cells co-transfected with MIF-3′-UTR and miR-152 mimics (mimic miR-152) showed reduced relative luciferase activity, compared with controls, whereas no change in activity was observed in samples co-transfected with MIF-mutant-3′-UTR and miR152 mimics **(A)** Cells co-transfected with MIF-3′-UTR and inhibitor miR-152 demonstrated an increase in relative luciferase activity **(B)** (^*^p < 0.05; ^***^p < 0.001). Luciferase reporter plasmids were constructed as previously described.

HeLa cells transfected with the MIF-3′-UTR and miR-152 mimic demonstrated a significantly reduced relative fluorescence activity, compared with the ones co-transfected with mimic negative control (nc) and MIF 3′-UTR (Figure 4A). In addition, there was an increase in relative fluorescence activity in HeLa cells co-transfected with the MIF 3′-UTR and miR-152 inhibitor, compared with ones co-transfected with inhibitor nc and the MIF 3′-UTR (Figure 4B). However, no change in the relative luciferase activity was observed in cells transfected with MIF-Mut-3′-UTR or normal controls (Figures 4A,B). The results above validated that 3′-UTR of MIF was a target of miR-152, and miR-152 downregulated the expression of MIF by binding MIF 3′-UTR, and inhibition of miR-152 reverses the reduced expression of MIF.

#### miR-152 Downregulated the Expression of MIF in Primary HRPTEs

**Figure 5:**
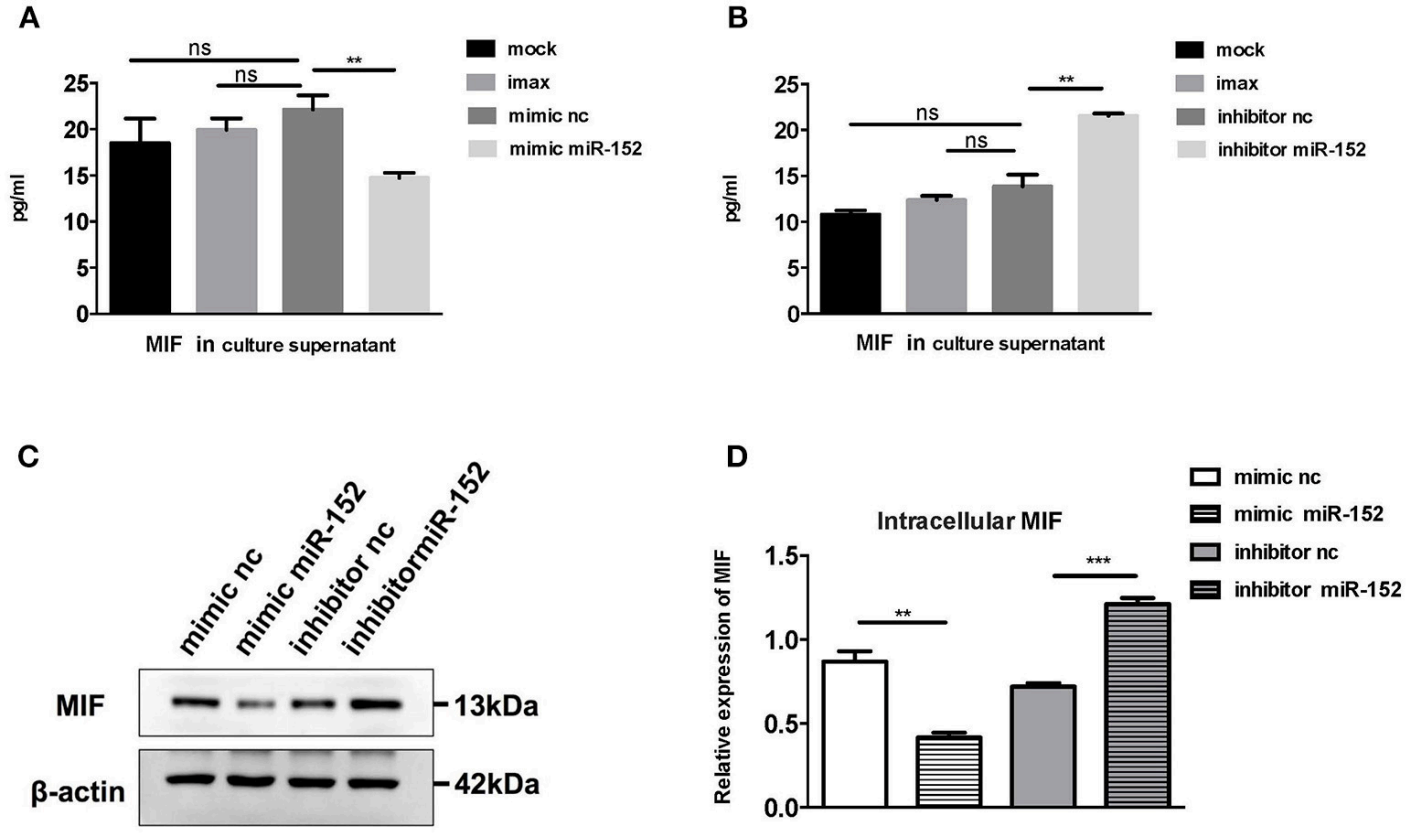
miR-152 decreased MIF expression in primary HRPTEs. Compared with controls, cells incubated with transfection reagent only (imax) and the ones transfected with normal controls (mimic nc and inhibitor nc), MIF levels in culture supernatant were reduced by miR-152 mimics **(A)** and upregulated by miR-152 inhibitor **(B)**. Similar results were observed with intracellular MIF expression by Western blotting **(C,D)**. (^**^P < 0.01 and ^***^P < 0.001).

To confirm the relationship between miR-152 and MIF, we examined the effect of miR-152 on MIF expression in primary HRPTEs. Twenty hours after the transfection of miR-152 mimics and inhibitors, intracellular MIF mRNA levels were significantly decreased in samples transfected with miR-152 and were elevated in samples transfected with inhibitor of miR-152. Similar upregulation and downregulation of MIF protein levels was observed in subsequent Western blotting (Figure 5).

#### miR-152 Was Downregulated in LN Tissue and its Expression Was Negatively Correlated With MIF Expression

**Figure 6:**
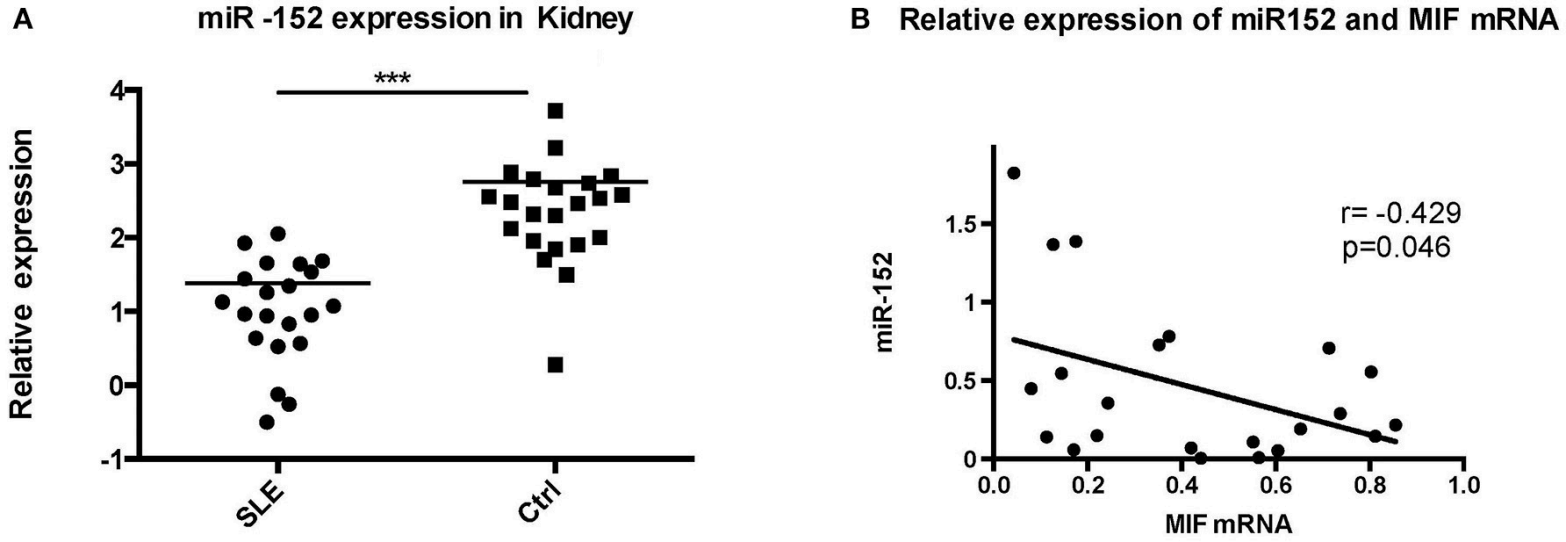
Significant decrease in miR-152 was observed in LN tissue **(A)** and a negative correlation was found between MIF mRNA levels and miR-152 levels **(B)** (^***^p < 0.001).

To further evaluate the relevance of the miR-152-mediated regulation of MIF in clinical specimens, we examined the levels of miR-152 in the LN tissue and the controls. RT-PCR showed miR-152 expression was much lower in LN tissue compared with the controls (Figure 6A; *p* = 0.0008). Furthermore, there was an inverse correlation between miR-152 and MIF levels in LN tissue when assessed by Pearson correlation analysis (Figure 6B; *r* = −0.429, *p* = 0.046).

#### The Expression of MIF Was Upregulated in the Proximal Tubular Cells of LN Tissue and MIF Expression Was Associated With Serum Creatinine Levels and 24 h Urine Excretion Levels

**Figure 7:**
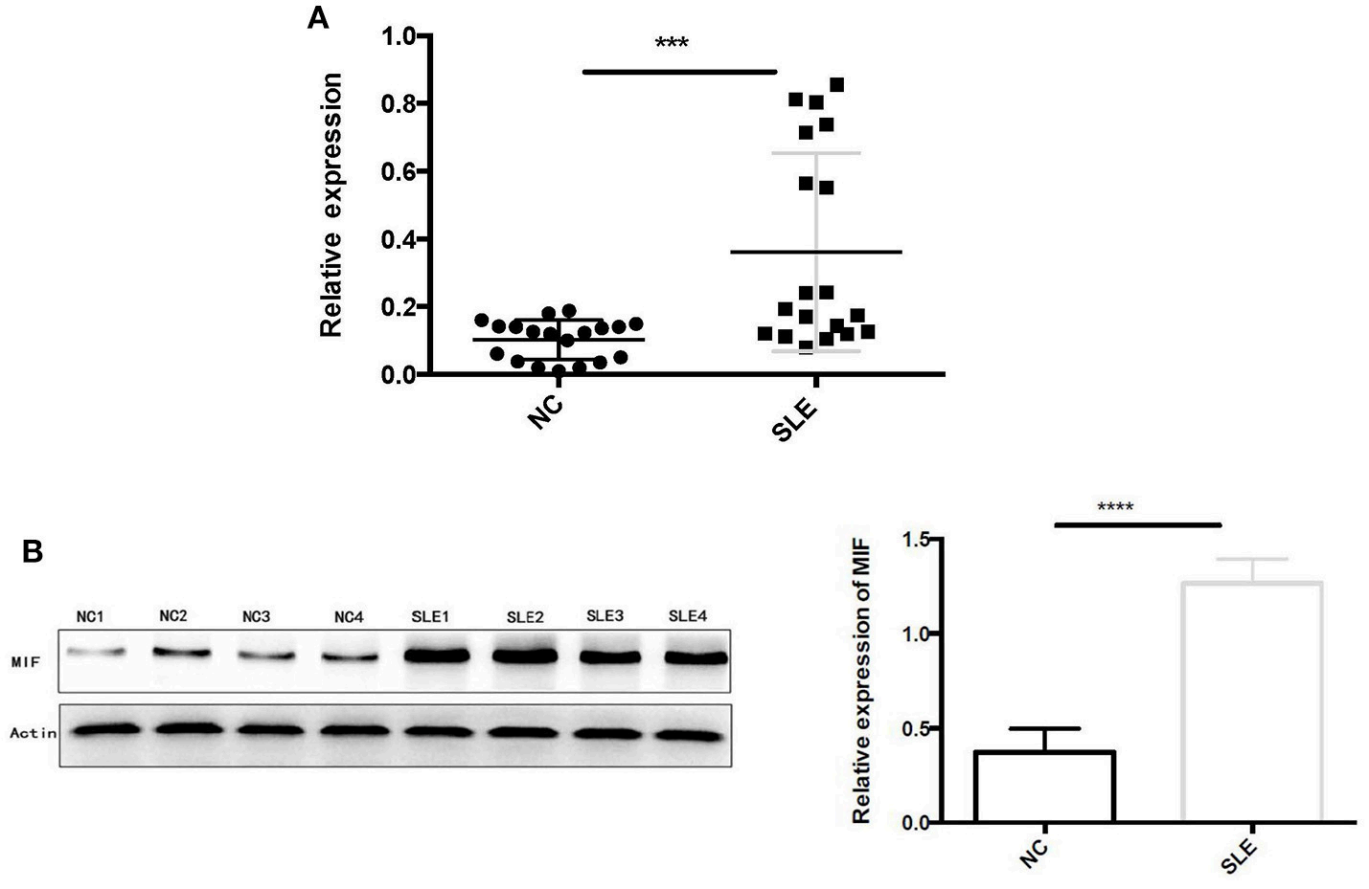
**(A)** Relative MIF mRNA levels in renal tissue of patients with LN and that in normal controls were demonstrated and compared. MIF mRNA expression is significantly higher in LN tissue compared with normal controls. **(B)** Western blot analysis of protein extracts of LN tissue and normal controls. Markedly elevated MIF protein levels were found in LN tissue (^***^P < 0.001 and ^****^P < 0.0001).

Using RT-PCR, we found out that MIF mRNA levels were elevated in LN tissue (Figure 7A), approximately 2 times that of normal controls (*p* = 0.0006). In addition, intracellular MIF protein levels were significantly elevated in LN patients, about 2 times higher than that of controls (*p* < 0.001; Figure 7B), as demonstrated by Western blotting analysis.

**Figure 8:**
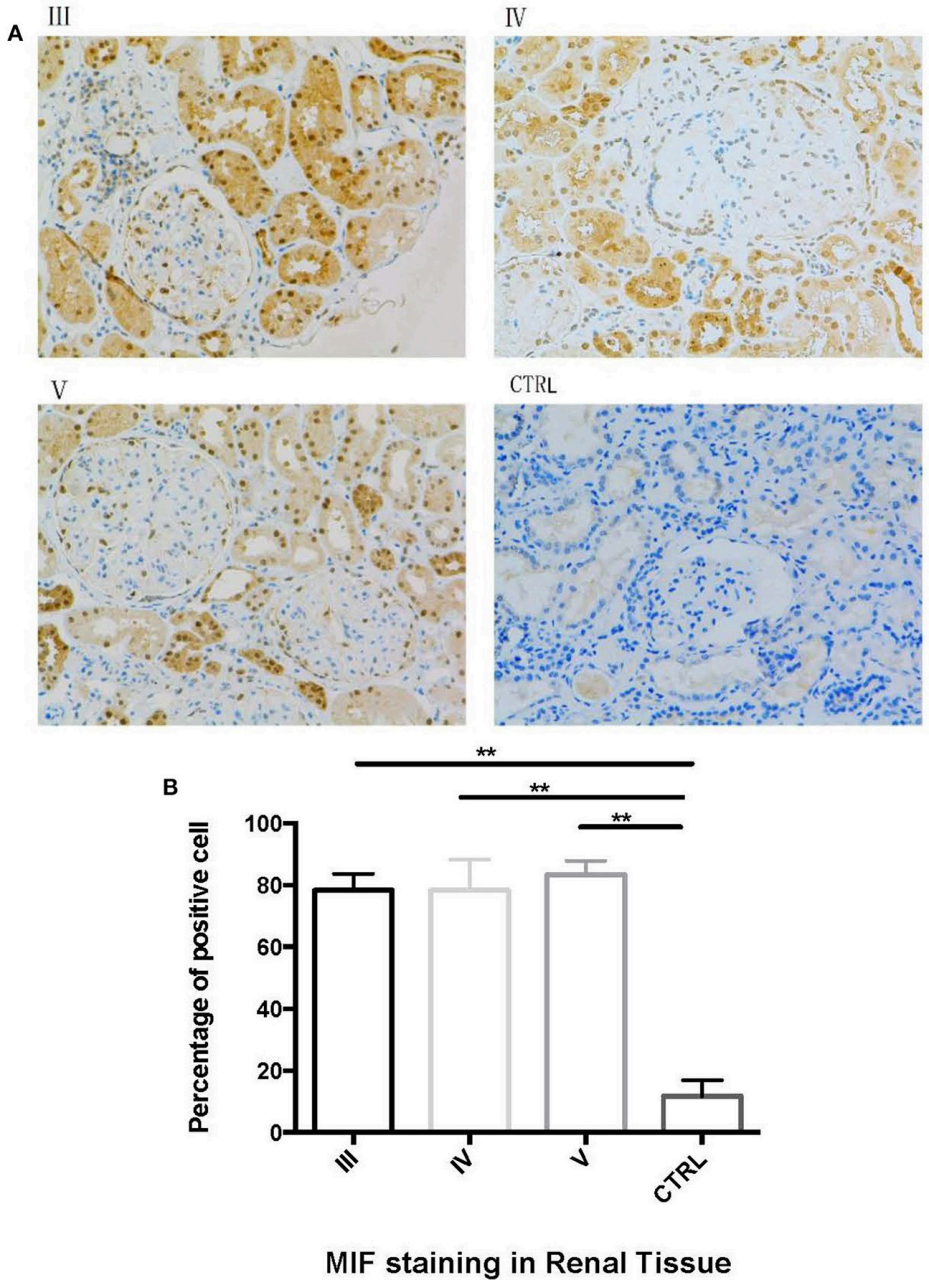
Immunohistochemical staining of MIF in different types of LN and in normal renal tissue. (**A**, 200x). Compared with control (CTRL in **A**), there was an aggregation of MIF staining in proximal renal tubular cells in class III (III in **A**), class IV (IV in **A**) and class V (V in **A**) LN. The percentage of MIF-staining cells were calculated and compared **(B)**. The percentage of MIF-positive cells were significantly higher than that of normal controls. (^**^P < 0.01).

Next, we performed immunohistochemical staining to locate the expression of MIF in LN tissue. In LN tissue, MIF staining was visible in the Bowman's capsule, glomerular parietal cells, and glomerular capillaries (Figure 8A). Compared with normal renal tissue, strong MIF staining was observed in LN tissue, especially in renal tubular region (Figure 8A). No difference of MIF expression was found among class III, IV, and V LN (Figure 8B). From a subcellular aspect, LN tissue demonstrated strong MIF staining in the cell nucleus and cytoplasm, whereas in normal renal tissue, MIF staining was found in the tubular lumen.

**Figure 9:**
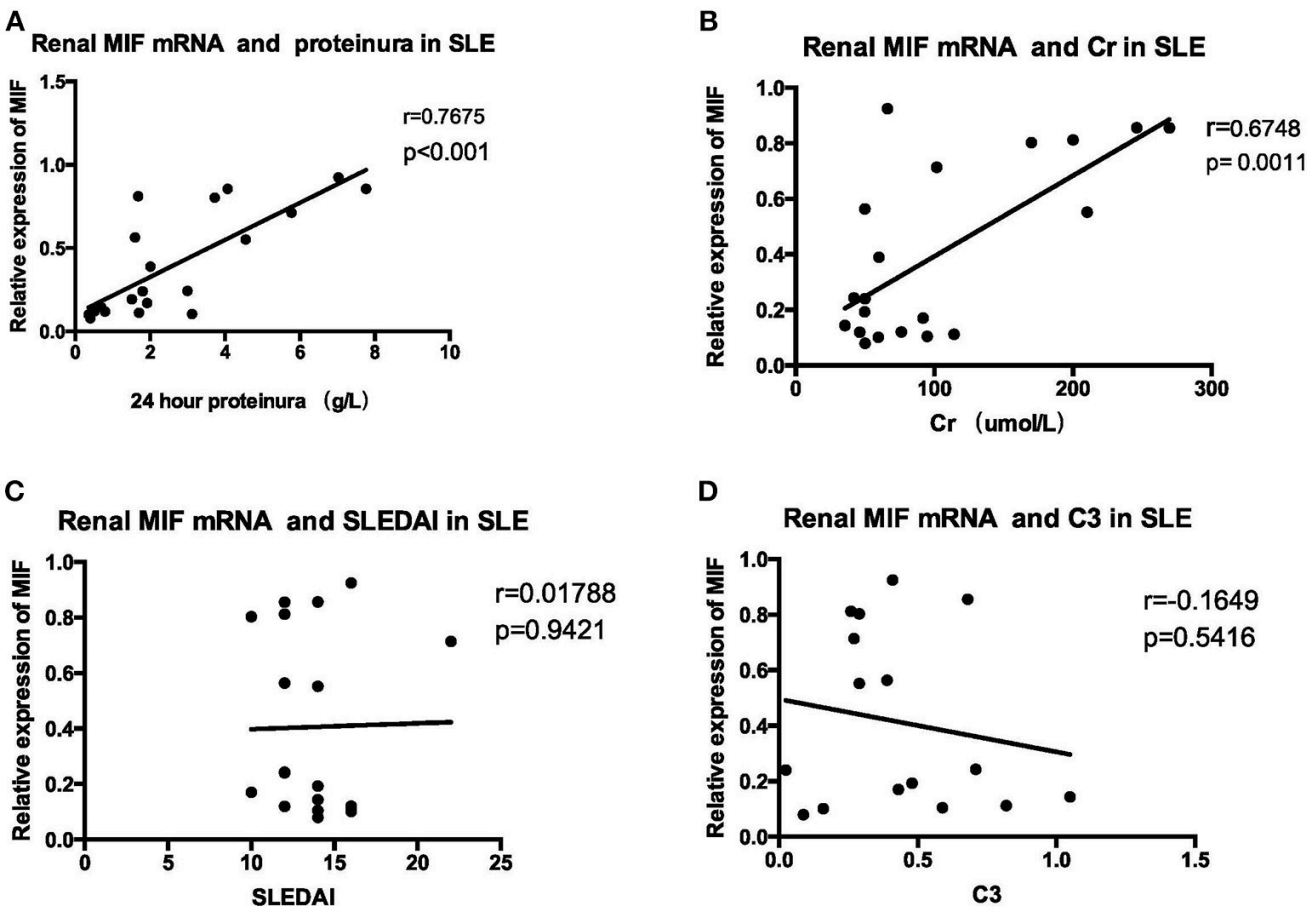
Correlation analysis between renal MIF mRNA expression and clinical and laboratory parameters **(A)** 24-h urine protein excretion levels **(B)** serum creatinine. **(C)** SLEDAI **(D)** serum complement levels. There was a positive correlation between renal MIF expression and 24 h urine protein excretion levels **(A)** and serum creatinine levels **(B)**, whereas no correlation was found between MIF expression and SLEDAI **(C)** or serum C3 levels **(D)**.

MIF mRNA levels were positively correlated with the severity of proteinuria, as represented by 24 h urine protein excretion levels (*r* = 0.7675, *p* < 0.001, Figure 9A), and serum creatinine levels (*r* = 0. 6748, *p* = 0.0011, Figure 9B). However, no correlation was found between MIF mRNA levels and SLEDAI scores (*r* = 0.01788, *p* = 0.9421, Figure 9C); nor was correlation found between serum complement C3 levels and MIF mRNA expression (*r* = −0.1649, *p* = 0.5416, Figure 9D).

### COL1A1, Downstream of MIF, Is Downregulated by miR-152

#### miRNA-152 Inhibited COL1A1 Expression in HRPTEs

**Figure 10:**
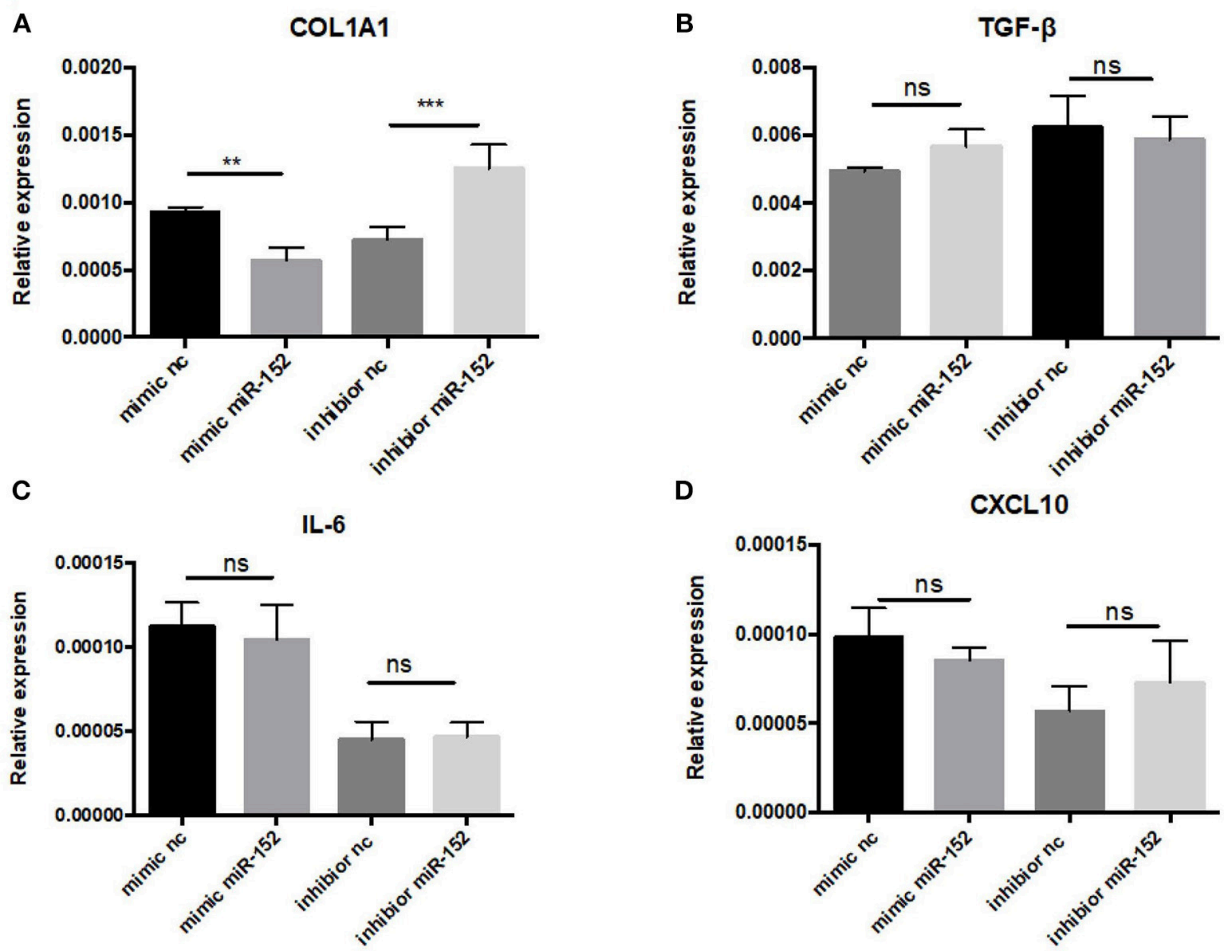
RT-PCR analysis of gene expression in primary HRPTEs treated with miR-152 mimic, miR-152 inhibitor, and controls. Compared with negative controls, the expression of COL1A1 was reduced by miR-152 mimics **(A)** and increased by miR-152 inhibitors, whereas there was no significant difference in TGF-β **(B)**, IL-6 **(C)** or CXCL10 **(D)** expression among all the groups. (^**^P < 0.01 and ^***^P < 0.001).

Markedly elevated MIF expression was observed in renal tubular epithelial cells (Figure 8). We therefore chose HRPTEs for further mechanistic investigation of the miR-152. miR-152 mimics and inhibitors were transfected to primary HRPTEs, and intracellular mRNA levels of COL1A1, TGF-β, interleukin-6 (IL-6) and CXCL10 were measured 24 h after transfection. Overexpression of miR-152 resulted in decreased COL1A1 mRNA expression, and inhibition of miR-152 led to an upregulation of COL1A1 expression. The intracellular mRNA levels of TGF-β, IL-6 and CXCL10 were unaffected (Figure 10).

**Figure 11:**
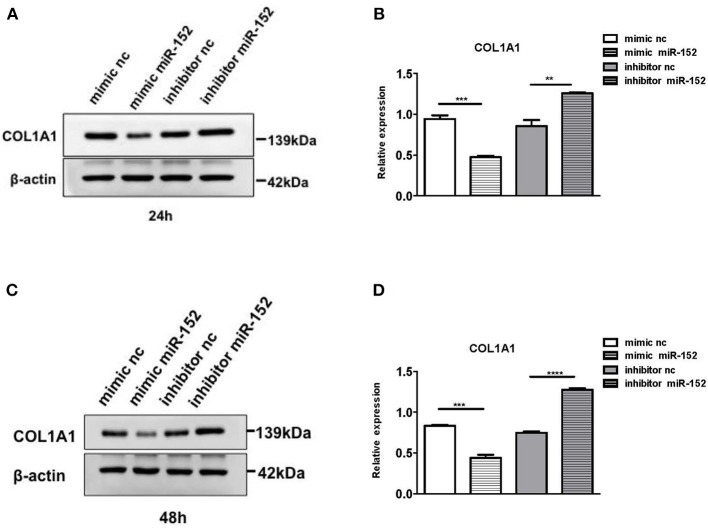
Western blot analysis of protein extracts of primary HRPTEs 24 h **(A,B)** and 48 h **(C,D)** after the transfection with miR-152 mimic (mimic miR-152), miR-152 inhibitor (inhibitor miR-152), and negative control RNA. Compared with negative controls (mimic nc or inhibitor nc), the expression of COL1A1 was significantly reduced by miR-152 mimic and increased by inhibitor miR-152 24 and 48 h after the transfection (^**^P < 0.01, ^***^P < 0.001, ^****^P < 0.0001).

We then performed Western blot analysis to confirm the effect of miR-152 on COL1A1 protein levels in primary HRPTEs. COL1A1 expression was measured 24 and 48 h after the transfection with miR-152 mimic and miR-152 inhibitor. COL1A1 expression was lessened 24 and 48 h following the transfection with miR-152 mimic (*p* = 0.0007 at 24 h and *p* = 0.0006 at 48 h, respectively) and it was upregulated 24 and 48 h following the transfection with miR-152 inhibitor (*p* = 0.0061 at 24 h, *p* < 0.0001 at 48 h; Figure 11).

#### Inhibition of MIF Production by siRNAs Downregulated COL1A1 Expression

**Figure 12:**
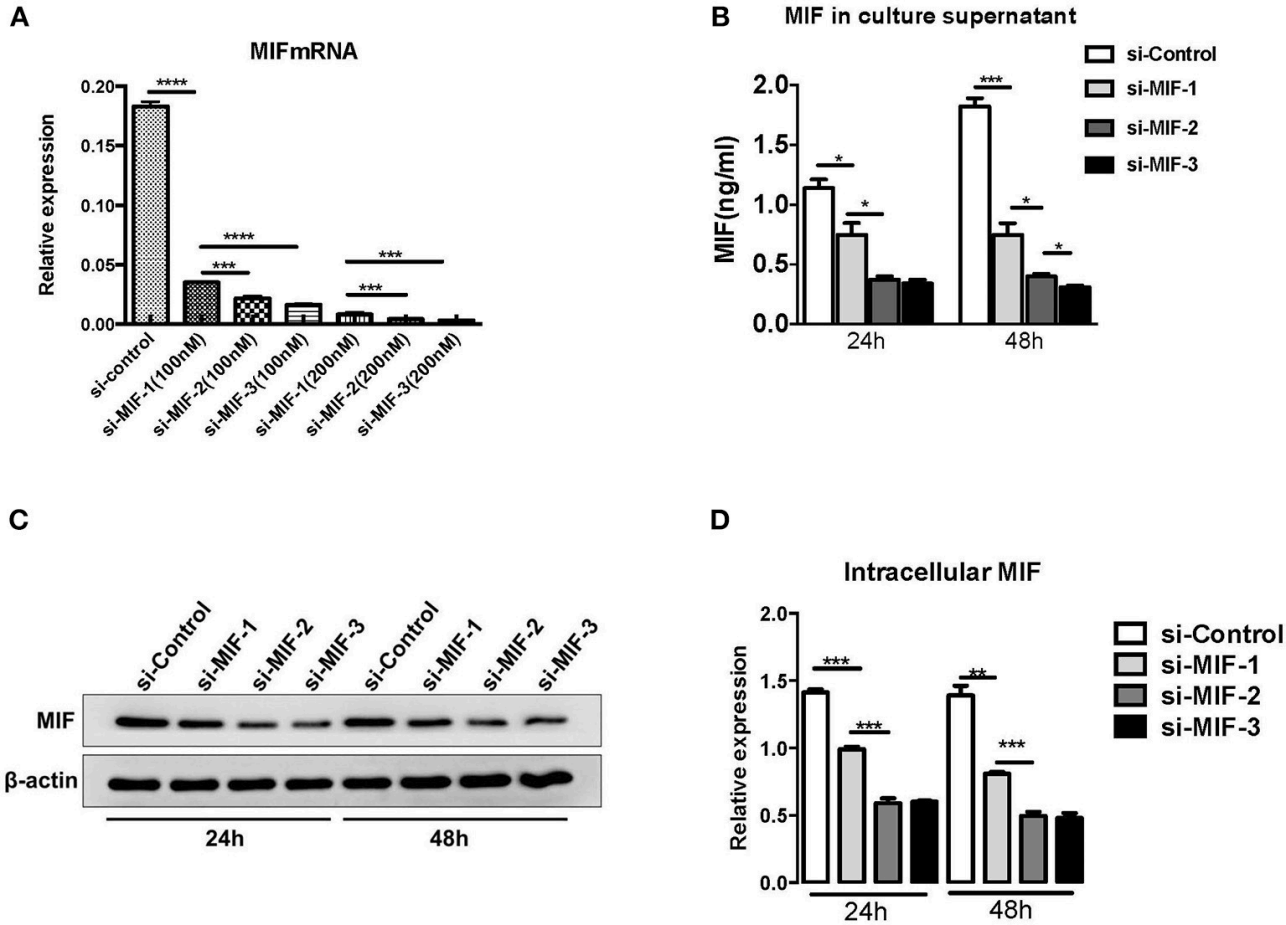
siRNAs targeting MIF in HRPTE were able to decrease MIF mRNA levels **(A)**, MIF protein levels in the culture supernatant **(B)** and intracellular protein levels **(C,D)**. (^*^P < 0.05, ^**^P < 0.01, ^***^P < 0.001, ^****^P < 0.0001).

**Figure 13:**
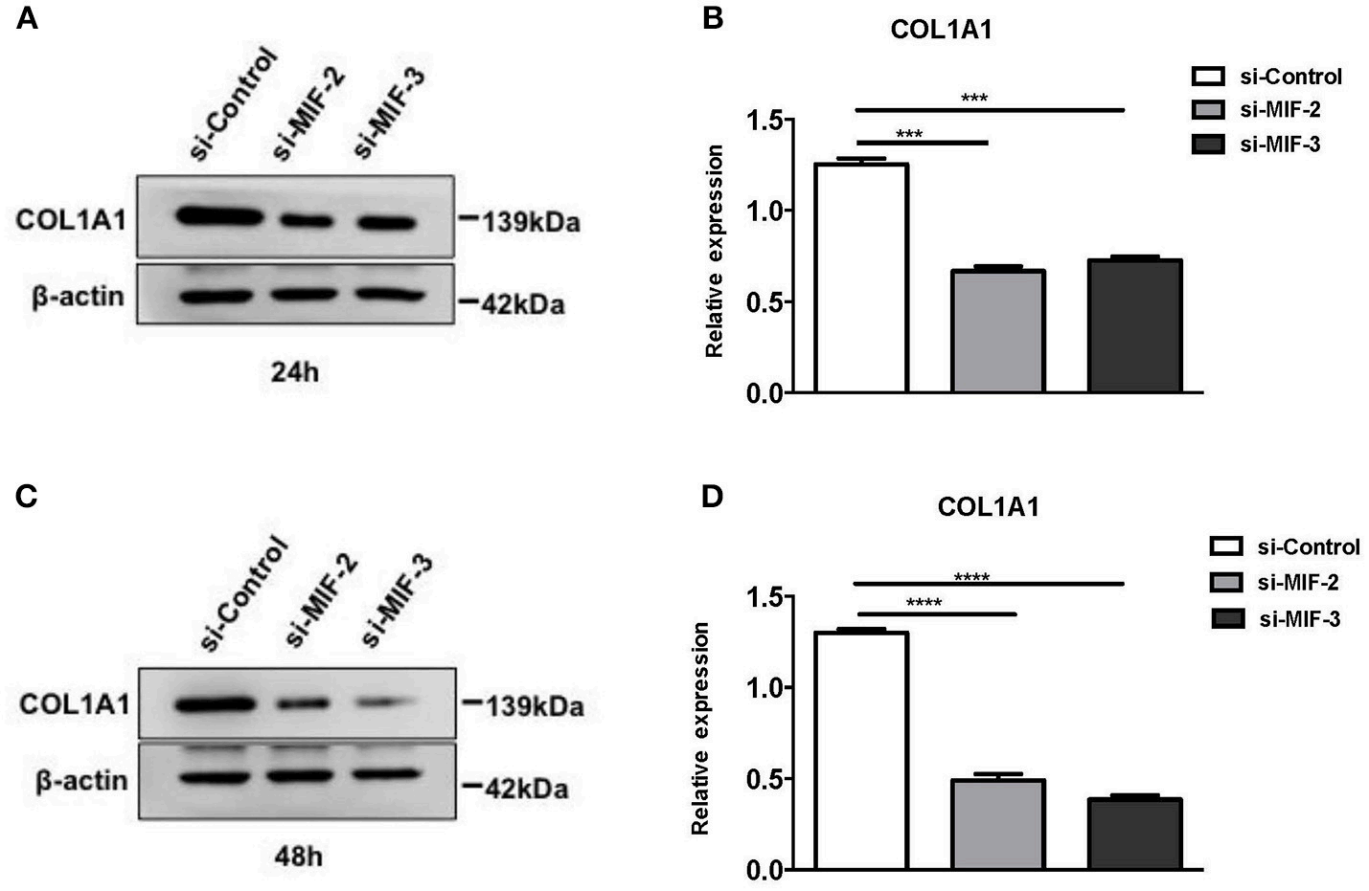
Western blot analysis of protein extracts of primary HRPTEs after transfection with siRNAs targeting MIF. Compared with negative control (si-control), the downregulation of COL1A1 was observed in HRPTEs treated with si-MIF-2, and si-MIF-3 24 **(A,B)** and 48 h **(C,D)** after transfection. (^***^P < 0.001 and ^****^P < 0.0001).

siRNAs were used to knock down MIF expression in *in vitro* cell lines for further investigation of the function of miR-152. We first designed three siRNAs consisting of different complementary base pairs that specifically targets MIF mRNA. The siRNAs, namely si-MIF-1, si-MIF-2, si-MIF-3, as well as the negative control si-control, were then transfected to HRPTEs. MIF expression was measured with RT-PCR and Western blotting after 24 and 48 h of incubation. Compared with si-control, si-MIF-1, si-MIF-2, and si-MIF-3 were able to reduce the expression level of MIF mRNA to different degrees (Figure 12). And si-MIF-2 and si-MIF-3 showed better knockdown efficiency and were therefore selected for follow-up experiments.

si-MIF-2, si-MIF-3, and negative control si-control were transfected into primary HRPTEs, and the COL1A1 expression levels were measured using Western blotting 24 and 48 h after the transfection, respectively, (Figure 13). Compared with si-control, si-MIF-2 and si-MIF-3 both significantly decreased the expression of COL1A1 24 and 48 h after the transfection.

#### COL1A1 Expression Was Upregulated in LN Tissue and Was Positively Correlated With MIF and Negatively Correlated With miR-152.

**Figure 14:**
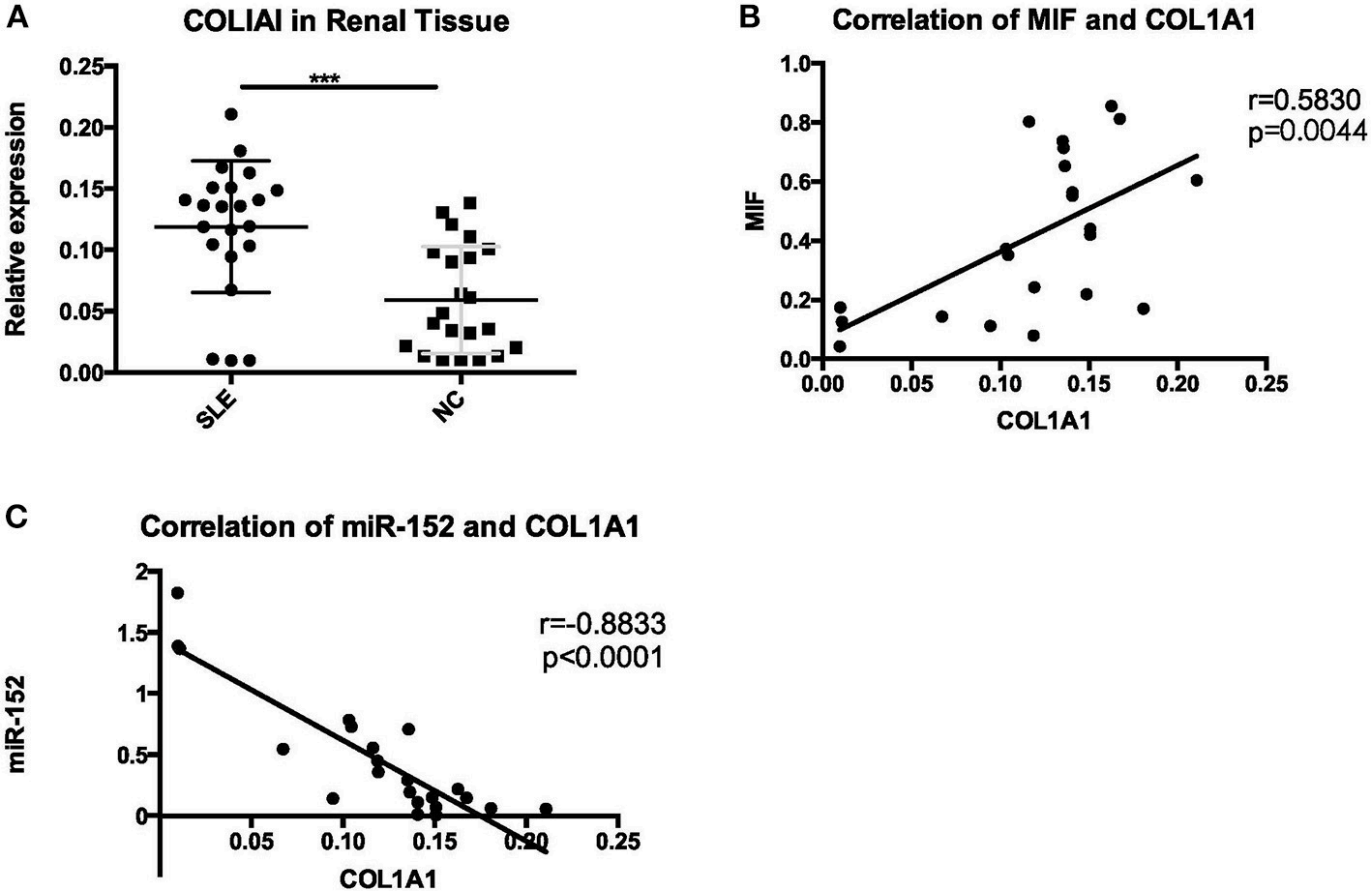
A significant increase of COL1A1 was detected in renal tissue of LN **(A)**, positive correlation with MIF **(B)**, and negative correlation with miR-152 **(C)** (^***^p < 0.001).

COL1A1 mRNA expression in LN tissue was significantly higher than that in controls (*p* = 0.0002) (Figure 14A). Then we performed a correlation analysis and found that there was a positive correlation between COL1A1 expression and MIF levels (*r* = 0.5830, *p* = 0.0044; Figure 14B). Besides, COL1A1 levels were negatively correlated with miR-152 (*r* = −0.8833, *p* < 0.0001; Figure 14C).

## Discussion

In this study, we chose to evaluate the expression of miRNAs in renal biopsy samples from LN patients and found that renal miR-152 expression was reduced in LN tissue compared with controls. We first used computational prediction and found that miR-152 may be the possible miRNA targeting MIF. Then we confirmed MIF a target of miR-152 by using a luciferase-based reporter assay. Based on the immunohistochemistry findings that there was an elevated MIF expression in renal tubular epithelial cells, we used HRPTEs for further investigation and we identified that miR-152 downregulated the expression of MIF through binding a sequence within the 3′-UTR of MIF.

miR-148/152 is a family of miRNAs that is extensively studied in recent years and the expression is found to be altered in both stem cells and tumor cells (20). In renal tissue, Ning et al. reported that miR-152 overexpression was able to downregulate collagen expression in TGF-β-incubated tubular epithelial cell line HK-2 (21). miR-152 is also involved in immunologic reactions. Liu et al. reported that miR-148/152 in C57BL/6 mice acted as a negative regulator for innate immune response and antigen-presenting function of dendritic cells by inhibiting the production of cytokines such as IL-12, IL-6, TNF-α, and LPS-induced upregulation of MHC class II expression and DC-initiated Ag-specific T cell proliferation by targeting calcium/calmodulin-dependent protein kinase II (CaMKII) (22). In addition, miR-152 was found elevated in the peripheral blood mononuclear cells (PBMCs) of SLE patients (7), suggesting its possible involvement in the pathogenesis of SLE.

MIF acts as an upstream regulator of innate immunity that influences the direction of the ensuing adaptive response (23). Previous studies showed that MIF levels were significantly elevated in SLE patients (15, 24), MIF expression was associated with SLE disease damage (SLICC/ACR index) (16) and urinary MIF/creatinine ratio was also linked to SLE disease activity (25).

MIF can be synthesized by different cells in kidney, such as podocytes, mesangial cells, tubular epithelial cells and endothelial cells. In LN, MIF expression was upregulated in glomerular endothelial and epithelial cells, and its expression was most evident at the basal aspect of proximal tubular epithelial cells (26, 27). Consistent with previous studies, we also found elevated MIF levels in LN tissue, especially in the tubular epithelial cells. In LN patients, renal MIF levels correlated with leukocyte infiltration, tissue damage and the impairment of renal function (26). These observations were also supported by experimental data in lupus-prone mice. MIF expression was elevated in the skin and kidney lesions of MRL/lpr mice prone to SLE, and *Mif*^−/−^mice have prolonged survival and attenuated skin and kidney lesions and urinary protein excretion levels when intercrossed into this spontaneous lupus strain (27). Small molecule MIF antagonists also resulted in reduced glomerulonephritis and interstitial inflammation, lower levels of CD74+ and CXCR4+ leukocyte recruitment and decreased circulating TNF-α (28). Together, these data have supported the ongoing clinical testing of an anti-MIF receptor antibody in LN (29).

In renal tissue, MIF and COL1A1 expression were both elevated in unilateral ureter obstruction (UUO) rats and in ischemia-reperfusion injury (IRI) rats (30). Our study also showed a positive correlation between MIF levels and COL1A1 expression in LN tissue. Studies have found that COL1A1 may be involved in the interstitial fibrosis that is related to progressive renal dysfunction (31) and its expression has been found associated with renal chronicity indices at the time of biopsy (32), although collagen type I expression in renal tubular epithelial cells might not directly contribute to collagen deposition in renal tissue (33, 34). In this study, we detected a similar correlation between COL1A1 expression and chronicity indices on renal biopsy.

COL1A1 expression may also be a sign of epidermal-mesenchymal transition (EMT) of injured renal tubular cells (34), a mechanism which leads to fibrosis of renal tissue and subsequent decline of renal function (35). In aristolochic acid (AA)-induced renal tubular epithelial cells, there was increased expression of MIF, collagen, α-smooth muscle actin (α-SMA) and decreased E-cadherin expression. The effect was inhibited by MIF antagonist ISO-1, suggesting the role of MIF as a potential upstream mediator of EMT that results in matrix deposition and subsequent renal fibrosis (36). We also reported a positive correlation between MIF expression and COL1A1 expression in LN tissue in this study. Therefore, we hypothesize that MIF-mediated EMT may be a possible mechanism of chronic renal injury in LN tissue, though more research is warranted to investigate the exact role of MIF in the pathogenesis of tubulointerstitial damage of LN.

Of note, the study by De la Cruz-Mosso et al. (37) demonstrated that PBMCs from patients with active SLE flare showed higher levels of TNF-α and IL-6, compared with that of patients in remission. In addition, MIF increased the secretion of IL-6, IL-8 and prostaglandin E2 in a dose-dependent manner (38). Although the correlation between IL-6 levels and MIF expression has been reported in multiple studies, in our study miR-152 did not appear to affect the IL-6 expression levels in renal proximal tubular cells, a finding that merits further mechanistic investigation of the role of miR-152 in LN. In addition, contrary to our conjecture, we did not observe downregulation of TGF-β expression in HRPTEs transfected with miR-152 agonist. Previous findings indicate that MIF may function as a mediator of TGF-β-induced fibrogenesis. Thus, we infer that miR-152 may inhibit pathways downstream of TGF-β, rather than direct downregulation of TGF-β synthesis. However, further investigations are necessary to validate this speculation.

Taken together, we demonstrate that miR-152 inhibits the renal damage in patients with LN by targeting MIF and reducing the expression of COL1A1. There also is a negative correlation between miR-152 expression and chronicity indices on renal biopsy, 24 h urine protein excretion levels and serum creatinine levels. This study has represented a novel association between miR-152 and LN. Yet, many questions still remain, especially about how miR-152 attenuates the tubulointerstitial lesion in LN.

## Author Contributions

YS and LLU contributed conception and design of the study. JZ and RG performed the experiments and analyzed the data. YT provided intellectual support, and contributed reagents and analytic tools for the study. RB and LLE provided guidance on the analysis of the data. JZ and RG drafted the manuscript. QF, JC, and LW revised the manuscript. All authors read and approved the submitted version.

### Conflict of Interest Statement

The authors declare that the research was conducted in the absence of any commercial or financial relationships that could be construed as a potential conflict of interest.

## Abbreviations

LN: lupus nephritis
MIF: macrophage migration inhibitory factor
HRPTE: human renal proximal tubular epithelial cell
PBMC: peripheral blood mononuclear cell
SLEDAI: Systemic Lupus Erythematosus Disease Activity Index
3′-UTR: 3′-untranslated region
EMT: epidermal-mesenchymal transition
α-SMA: α-smooth muscle actin.
