# Supplementary material for: miR-152 Attenuates the Severity of Lupus Nephritis Through the Downregulation of Macrophage Migration Inhibitory Factor (MIF)-Induced Expression of COL1A1
Source: Front Immunol. 2019 Feb 6;10:158. doi: 10.3389/fimmu.2019.00158 (PMC6372555; doi:10.3389/fimmu.2019.00158)
Supplement: Supplementary file 1 [file Table_1.DOCX]

**Supplementary Table 1. Clinical features of patients with lupus nephritis and patients with renal cell carcinoma (healthy controls).**

|  | Control (N=20) | LN(N=22) |
| --- | --- | --- |
| Male/Female | 12/8 | 4/18 |
| Age (years) | 42 ± 11 | 29.7 ± 10.4 |
| Disease duration (month) | - | 60.5 ± 54.2 |
| SLEDAI score | - | 13.5±2.7 |
| C3 level (mg/ml) | - | 0.46 ± 0.27 |
| Serum creatinine (umol/L) | - | 82.7 ± 55.6 |
| 24-hour urinary protein excretion level (gram) | - | 2.82 ± 2.02 |
| Activity Index |  | 7.3 ± 4.1 |
| Chronicity Index |  | 3.3 ± 2.2 |
| Stage* | I-II | - |

*Stage of renal cell carcinoma

‘—’ indicates ‘not applicable’.

**Supplementary Table 2. Demographic, clinical, and laboratory findings of the LN patients.**

| Classification^#^ | Class III | Class IV | Class V | Class V+III | Class V+IV | All |
| --- | --- | --- | --- | --- | --- | --- |
| Number of patients | 4 | 3 | 5 | 4 | 6 | 22 |
| Age | 23.6±5.2 | 28.8±4.4 | 32.0±11.7 | 33.4±13.5 | 29.8±7.9 | 29.7 ± 10.4 |
| SLEDAI | 14.0±2.0 | 12.6±1.9 | 13.2±2.0 | 13.0±1.0 | 14.2±4.4 | 13.5±2.7 |
| 24h-urinary protein excretion level (gram) | 1.34±0.57 | 3.48±0.77 | 2.54±2.67 | 1.7±1.43 | 4.12±1.73 | 2.73±2.0 |
| Serum creatinine (umol/L) | 70.0±25.7 | 123.0±62.9 | 67.8±20.6 | 103.8±95.6 | 66.6±19.7 | 82.7±55.6 |
| C3(mg/ml) | 0.41±0.25 | 0.55±0.2 | 0.50±0.42 | 0.42±0.25 | 0.46±0.17 | 0.46±0.27 |
| Disease duration (median months) | 78 | 32 | 35 | 56 | 77 | 48 |
| AI | 6.7±3.4 | 8.6±4.5 | 4.4±3.0 | 6.7±3.5 | 9.8±3.4 | 7.3±4.1 |
| CI | 3.5±2.2 | 3.3±1.2 | 2.6±2.4 | 3.0±2.0 | 3.8±2.1 | 3.3±2.2 |

^#^The 22 LN patients were classified based on ISN/RPS classification.
